# Paid family leave to promote breastfeeding: catching policy up to the science

**DOI:** 10.1038/s41390-026-05341-5

**Published:** 2026-07-24

**Authors:** Diana Montoya-Williams, Scott A. Lorch

**Affiliations:** 1 Division of Neonatology, Children’s Hospital of Philadelphia, Philadelphia, PA USA; 2 CHOP PolicyLab, Children’s Hospital of Philadelphia, Philadelphia, PA USA; 3 University of Pennsylvania Perelman School of Medicine, Philadelphia, PA USA; 4 Pediatric Policy Council, Washington, DC USA; 5 Academic Pediatric Association, McLean, VA USA; 6 American Pediatric Society, The Woodland, TX USA; 7 Association of Medical School Department Chairs, McLean, VA USA; 8 Society for Pediatric Research, The Woodlands, TX USA

## Abstract

**Abstract:**

Breastfeeding is increasingly recognized as a powerful biologic intervention associated with positive infant and maternal health outcomes. Emerging evidence continues to expand our understanding of the mechanisms through which human milk promotes health, including via immune system development and modulation of antimicrobial resistance. Yet despite the robust scientific evidence supporting breastfeeding as critical to public health, many families in the US face structural barriers to achieving their breastfeeding goals. In the US, paid family leave has been strongly associated with both the initiation of breastfeeding and its duration. Paid family leave has also been associated with improved maternal and infant health, including reduced infant mortality. These findings suggest that paid family leave should be viewed not solely as a labor policy, but as a maternal-child health policy. The US remains one of the few high-income nations without a federal paid family leave program, relying instead on a fragmented patchwork of state policies. State experiences demonstrate that paid family leave can be implemented without major labor market disruption, while public support for a national program remains high. As breastfeeding science continues to advance, federal paid family leave represents overdue public health infrastructure necessary to translate evidence into population-level health improvements.

**Impact:**

- Paid family leave is one of the most effective and well-supported policy mechanisms for promoting breastfeeding success and improving maternal and infant health. This article synthesizes emerging breastfeeding science, including new evidence regarding human milk’s influence on neonatal immunity and antimicrobial resistance. We then link breastfeeding research with research on the policy that most supports widespread breastfeeding at a population level: paid family leave. Translating the benefits of breastfeeding into improved public health will require national policies that enable families to achieve breastfeeding goals. Federal paid family leave is among the most evidence-based policy strategies to achieve such goals.

There is a vast yet continuously growing evidence base linking receipt of maternal milk with positive health outcomes across infancy, childhood, and adulthood.^1,2^ Recent advances in breastfeeding science continue to deepen our understanding of how profoundly early nutrition shapes health. For instance, receipt of maternal milk has been well-linked to the development of neonatal immunity not only via passive immunity and immunoglobulin transfer but also by actively modulating the developing infant immune system.^3^ The paper by Mathkor and colleagues in this issue of *Pediatric Research* synthesizes the growing body of evidence around yet another pathway through which breastfeeding promotes health: by positively influencing the neonatal “resistome” and the genes controlling antimicrobial resistance in babies.^4^

With every paper, we learn more ways in which receipt of human milk can be viewed as a powerful biologic intervention with measurable effects on immune development, infection risk, chronic disease, and neurodevelopmental outcomes.^2,5^ Yet while the evidence base supporting breastfeeding continues to expand, the United States has failed to uniformly implement the policies most likely to help families achieve breastfeeding goals. One such policy with the strongest evidence base is paid family leave.^6^ Although breastfeeding initiation rates in the United States are relatively high, sustaining breastfeeding—particularly exclusive breastfeeding—remains challenging for many families once parents return to work.^7^ Paid family leave has repeatedly been associated with increased breastfeeding initiation, longer breastfeeding duration, and higher likelihood of breastfeeding throughout the first year of life.^8–11^

Importantly, the benefits of paid leave appear to extend beyond breastfeeding outcomes alone. Studies of state-level paid family leave policies have found reductions in infant hospitalizations overall^12^ and admissions for common respiratory infections due to respiratory syncytial virus.^13^ Importantly, international data showing that paid family leave is associated with reduced infant mortality has also been recapitulated here.^14^ A study of the California paid family leave policy suggested that paid family leave may be particularly important in reducing post-neonatal mortality or deaths occurring after the first month of life.^15^ This is a period of time during which parental presence and capacity may substantially decrease the drivers of infant mortality such as suboptimal breastfeeding, increased infection risks, and poor adherence to safe sleep practices.

The dividends of paid family leave are also not limited to infants. Policies that support breastfeeding and parental presence during the postpartum period may also improve maternal health outcomes. Maternal health benefits associated with breastfeeding are well-known and include reductions in maternal cardiovascular disease, metabolic disease, and some cancers.^5,16^ However, paid family leave itself has been linked to lower rates of severe maternal morbidity and mortality during the critical first year after delivery.^17^ Evidence suggests that paid family leave and breastfeeding support may reduce adverse maternal mental health outcomes, including postpartum depression and severe psychological distress.^11,18^ In this sense, paid family leave should not be viewed solely as a labor policy or even solely as a child health policy. It is a maternal-child health policy with potential multigenerational impact.

Despite this robust evidence base, the United States remains one of the few high-income countries without a federal paid family leave policy. Instead, families face a fragmented patchwork of state-level policies that vary substantially in duration, eligibility, wage replacement, and funding structure—leaving support dependent largely on where a family lives and who employs them.^19^ Debates surrounding paid family leave implementation frequently center on financing mechanisms. Funding approaches differ across states, with many programs relying on payroll tax models involving employee contributions, employer contributions, or combinations of both.^19^ Yet despite longstanding concerns about economic consequences, evidence from states that have implemented paid family leave policies has not demonstrated adverse labor market effects. To the contrary, many studies suggest minimal disruption to employers and potential benefits for workforce retention and employee well-being.^14,20^

Importantly, momentum toward paid family leave is growing even within politically diverse states. As of June 2026, fourteen states and the District of Columbia have established mandatory paid family leave programs. An additional nine states have instead adopted a voluntary system whereby paid family leave insurance can be purchased.^19^ Despite the wide variability in implementation, these efforts demonstrate that paid family leave is not exclusively a partisan issue but increasingly recognized as a practical investment in families and population health. Still, while state-level progress is encouraging, the patchwork approach will inevitably leave many families behind.

The closest the United States has come to a federal paid family leave framework occurred during the COVID-19 pandemic, when temporary federal leave protections were enacted through emergency legislation.^21^ Notably, public support for these policies is remarkably high. Surveys conducted during and after the pandemic demonstrated that nearly 80% of registered voters in the US supported a national paid family and medical leave program, including majorities across political affiliations.^22^ In other words, Americans continue to broadly support the importance of ensuring that families can care for newborns and loved ones without risking economic catastrophe.

The science is thus clear: Breastfeeding has substantial benefits for both mothers and infants, and paid family leave is one of the most effective policy mechanisms to promote breastfeeding success. State experiences show us paid family policies can be implemented without major labor market harm. Public support for such policies has been and remains high. What remains absent is not evidence, feasibility, or public will—but national action. If the US is serious about translating maternal-child health science into population health improvement, then federal paid family leave should no longer be viewed as an aspirational policy. It should be viewed as overdue public health infrastructure.
